# Influence of microwave-assisted dehydration on morphological integrity and viability of cat ovarian tissues: First steps toward long-term preservation of complex biomaterials at supra-zero temperatures

**DOI:** 10.1371/journal.pone.0225440

**Published:** 2019-12-04

**Authors:** Pei-Chih Lee, Daniella M. Adams, Olga Amelkina, Kylie K. White, Luigi A. Amoretti, Marinda G. Whitaker, Pierre Comizzoli

**Affiliations:** Smithsonian Conservation Biology Institute, National Zoological Park, Washington, District of Columbia, United States of America; Colorado State University, UNITED STATES

## Abstract

Ovarian tissue contains large pools of immature oocytes enclosed in primordial follicles, making it an attractive target for fertility preservation in female cancer patients, livestock and wild species. Compared to cryopreservation, desiccation and long-term storage of samples at supra-zero temperatures (using strategies inspired from small organisms to resist extreme environments) would be more cost-effective and convenient. The objective of the study was to characterize the influence of microwave-assisted dehydration on structural and functional properties of living ovarian tissues. While this method allows preservation of single cells (cat oocytes and sperm cells so far) using trehalose as the xeroprotectant, it has not been developed for multicellular tissues yet. Ovarian cortex biopsies were reversibly permeabilized, exposed to various concentrations of trehalose, and dried for different times using a commercial microwave under thermal control. Effective dehydration of samples along with proper trehalose retention were reached within 30 min of microwave drying. Importantly, the process did not affect morphology and DNA integrity of follicles or stromal cells. Moreover, transcriptional activity and survival of follicles were partially maintained following 10 min of drying, which already was compatible with storage at non-cryogenic temperatures. Present data provide critical foundation to develop dry-preservation techniques for long-term storage of living multicellular tissues.

## Introduction

Formation of primordial follicles occurs early in mammalian ovaries [1, 2]. These quiescent follicles are embedded in the ovarian cortex and become the source of recruited follicles and candidates for ovulation at each estrus cycle throughout the reproductive years [1, 3]. The high reproductive potential harbored by the pool of primordial follicles makes ovarian cortex tissue an appealing target for fertility preservation, particularly when mature oocytes or embryos cannot be collected [4, 5]. Ovarian tissue preservation is a favorable choice for young cancer patients before undergoing chemotherapy and for women who are not able to receive gonadotropin stimulation for oocyte retrieval due to diseases [5, 6]. Similar methods can also be applied to gamete rescue of genetically valuable animals, including domestic and wild species, to extend their genetic lifespan [7, 8].

The most common approach for ovarian tissue preservation is cryopreservation. Although once considered experimental, it is increasingly integrated into routine human fertility preservation [5]. Despite various degree of success reported in different species, morphological, cytological and molecular alterations are observed in cryopreserved tissue in many species studied, including mouse [9, 10], cat [11, 12], sheep [13], pig [14], cattle [14, 15], monkey [16], and human [17, 18]. Although cryopreserved biomaterials can be stably stored at cryogenic temperatures, the dependence on liquid nitrogen storage generates high demand on long-term maintenance and financial sustainability. Therefore, the accessibility, expandability and transportability of cryo-banks are limited especially in regions with low resources. Preservation of biomaterials at supra-zero temperatures under a dry state has emerged as a less detrimental and more economical alternative. The concept of dry-preservation is derived from natural anhydrobiosis that some invertebrate species utilize to survive extreme drought [19]. At the core of this strategy is the accumulation of intracellular trehalose, a disaccharide sugar that reaches a stable glass state and secures the embedded cellular content under dried conditions [20, 21]. As vertebrates lack trehalose synthesizing genes [22], mimicking that process in these species requires the delivery of trehalose directly into cells before drying (through membrane poration, endocytosis, engineered trehalose, or cell penetrating carriers) [23]. Desiccation in the presence of intracellular trehalose as protectant allows storage at non-cryogenic temperatures and hence eliminates the need and risk of maintaining and handling liquid nitrogen tanks [24].

Trehalose is a superior protectant because of its high glass transition temperature (T_g_) compared to other forms of sugar. Biomaterials can be stably maintained in trehalose glass as long as it is kept below T_g_ (the higher the concentration of trehalose, the higher the T_g_, and vice versa). In general, after exposing biomaterials to trehalose solution (usually below 1.5 M), there are two routes to achieve liquid-to-glass transition (a.k.a. vitrification). One is cryogenic vitrification that rapidly lowers the temperature below the T_g_ at lower trehalose concentrations. The other way is isothermic vitrification by elevating trehalose concentration through water removal from the solution without drastic change of temperatures. Multiple approaches are possible to reach the latter (spin drying [25] or vacuum drying [26], for instance). Among these techniques, microwave-assisted dehydration is fast and simple. It has been developed to uniformly desiccate samples through microwave-induced vibration and evaporation of water molecules [27]. Utilizing a commercially available SAM 255 microwave system, low intracellular water content can be reached in a controlled manner without marked elevation of temperature in the samples [27]. Using this drying method with trehalose as the ‘xeroprotectant’, promising results have been reported for dry-preservation of germinal vesicle and spermatozoa [28, 29]. However, this approach has yet to be tested in more complex biomaterials, such as multicellular tissues.

Our laboratory has been studying ovarian tissue cryopreservation for the last decade in the domestic cat. This species serves as a biomedical model not only for rare and endangered felids but also for human given their shared traits in ovarian anatomy and physiology [30, 31]. Living tissue recovery from cryogenic vitrification has been effectively evaluated by follicular/stromal morphology, DNA integrity, transcriptional activity, and survival [11]. Using this established system as a reference, we sought to develop microwave-assisted drying for living ovarian tissue samples. According to a Gordon-Taylor fit of trehalose-water data, the maximal moisture content allowed for sustaining glass state at 4°C is 0.1328 gram water over gram dried solute weight (gH_2_O/gDW) [32]. The same equation has been employed to select optimal drying time for germinal vesicles and spermatozoa under the assumption that T_g_ will not be altered significantly by the small mass contribution of the single cells [28, 29]. However, this cannot be applied to tissues that possess considerable mass and heterogeneous cell composition. Therefore, systematic evaluations are required to determine the dehydration degree suitable for tissue preservation and storage at supra-zero temperatures. The objective of the study was to characterize the influence of microwave-assisted dehydration after exposure to trehalose on morphology and viability of living ovarian tissues. Specifically, we explored the kinetics of water loss and trehalose retention over the course of microwave drying. We then further examined the effect of drying time, trehalose concentrations, and storage on tissue histology, DNA integrity, transcriptional activity, or follicle survival.

## Results

### General conditions for the microwave-assisted dehydration protocol

**Fig 1:**
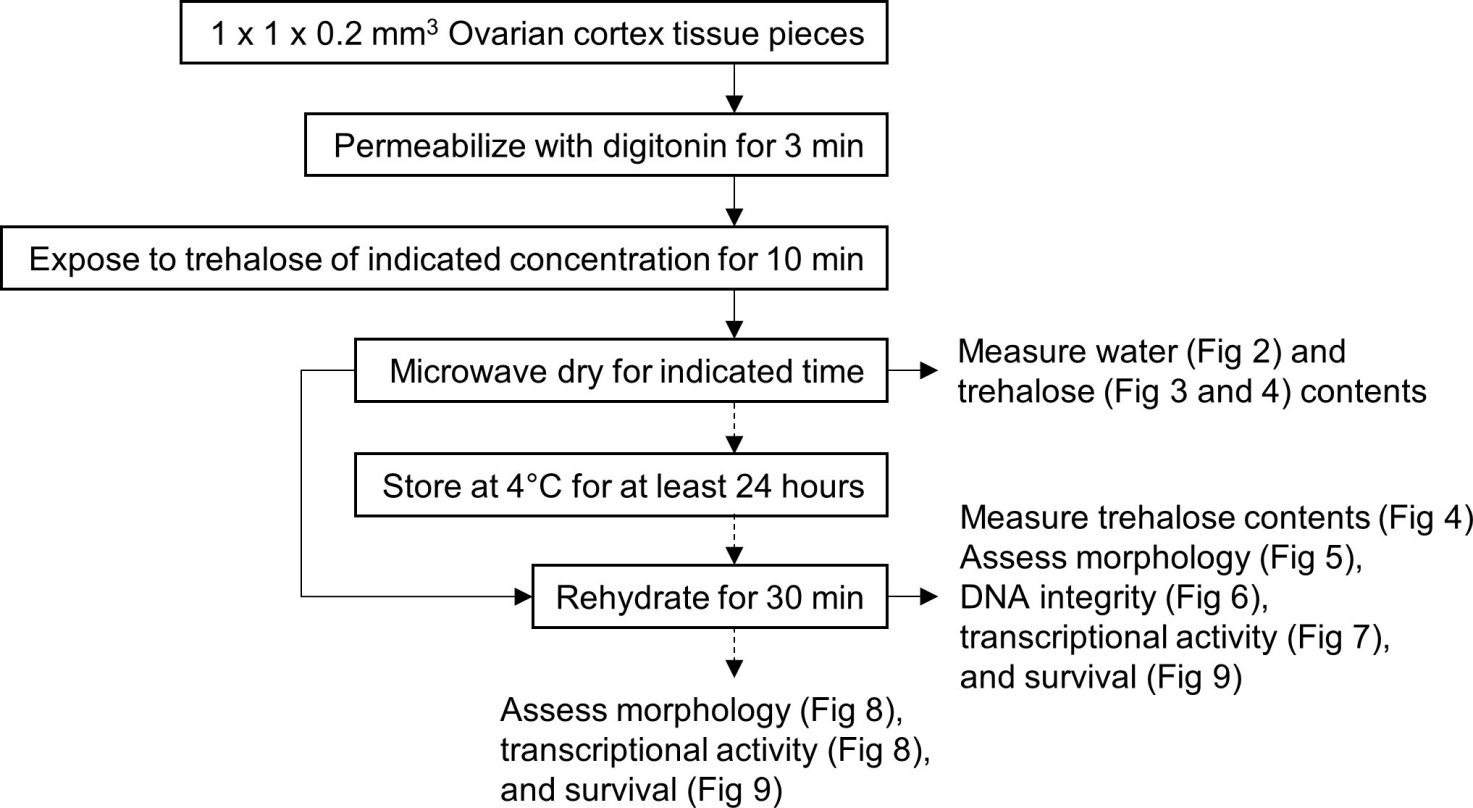
Flow chart of microwave-assisted dehydration procedure and evaluations. Ovarian tissue pieces were assessed after drying, after drying and immediate rehydration (solid arrows), or after storage and rehydration (dotted arrows).

Our microwave-assisted dehydration protocol was adapted from the one previously developed for individual cells [28, 29]. In brief, ovarian cortical tissue was dissected into 1 x 1 x 0.2 mm^3^ pieces, permeabilized with digitonin for 3 min, exposed to trehalose for 10 min, then microwave dried at 20% power with maximum temperature set to 40°C (Fig 1).

### Kinetics of water loss in tissues during microwave drying

Because microwave drying has not been tested for tissue preservation, we first needed to characterize the drying kinetics of ovarian biopsies. Trehalose-exposed tissue pieces were dried for up to 40 min, and water content was measured at 5-min intervals. Resulting drying curves were fitted to a logarithmic model (Eq (1)).

$$W_{t}=(W_{0}-W_{e})\exp\left(-kt\right)+W_{e}$$ (1)

wherein *W*_*t*_, *W*_*0*_, and *W*_*e*_ are water content at any given time (*t*) of drying, initial water content, and equilibrium water content, respectively, and *k* is the fitting parameter.

**Fig 2:**
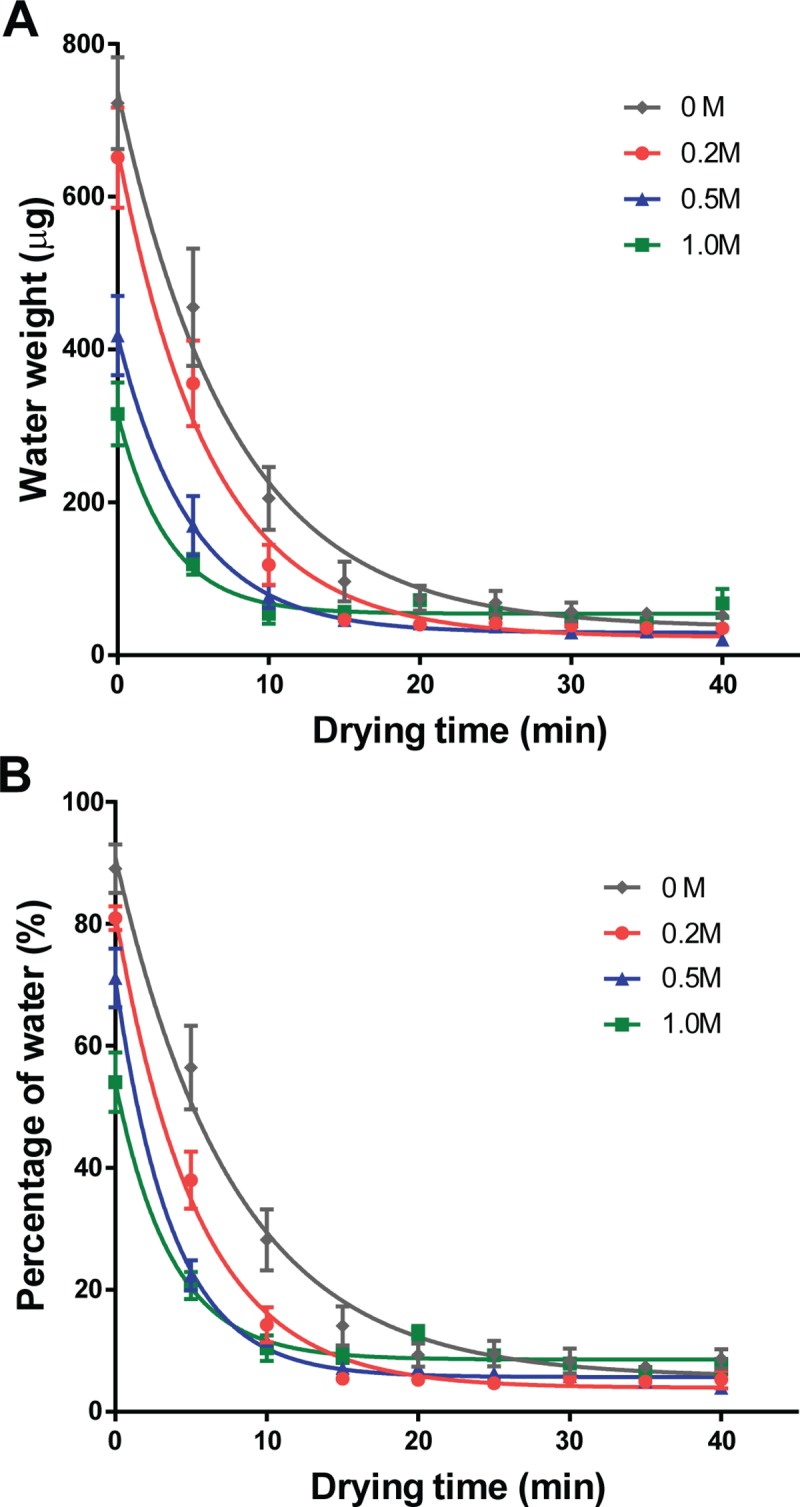
Kinetics of water content in tissues during microwave drying. Ovarian tissues were exposed to 0 M, 0.2 M, 0.5 M, or 1.0 M trehalose before microwaving for 0 to 40 min. Water contents were expressed as (A) water weight (μg) and (B) percentage of water weight over total tissue weight. Values are mean ± SEM. Drying curves were fitted to a logarithmic model Eq.(1).

At Time 0, each tissue piece exposed to buffer solution alone (0 M trehalose) contained 722 ± 60 μg of water, corresponding to 89.1 ± 4.0% of tissue weight (Fig 2). Water content was lower as the concentration of trehalose solutions increased (0.2 M: 651 ± 66 μg [81.0 ± 2.0%]; 0.5 M: 418 ± 52 μg [71.2 ± 4.8%]; 1.0 M: 316 ± 41 μg [54.1 ± 4.9%]; Fig 2) as a result of hyperosmotic conditions. In each solution, 68 to 84% of water evaporated within the first 10 min, then an additional 7 to 23% was gradually lost over the next 30 min to reach a steady low water content. The model predicted the equilibrium water content to be at 23.8 to 54.4 μg (3.9 to 8.6%; S1 Table). For the trehalose concentrations tested, water contents reached 95% confidence interval of *W*_*e*_ within 30 min of drying–a time that was kept for the following experiments.

### Effect of dehydration and rehydration on trehalose content and gross tissue morphology

**Fig 3:**
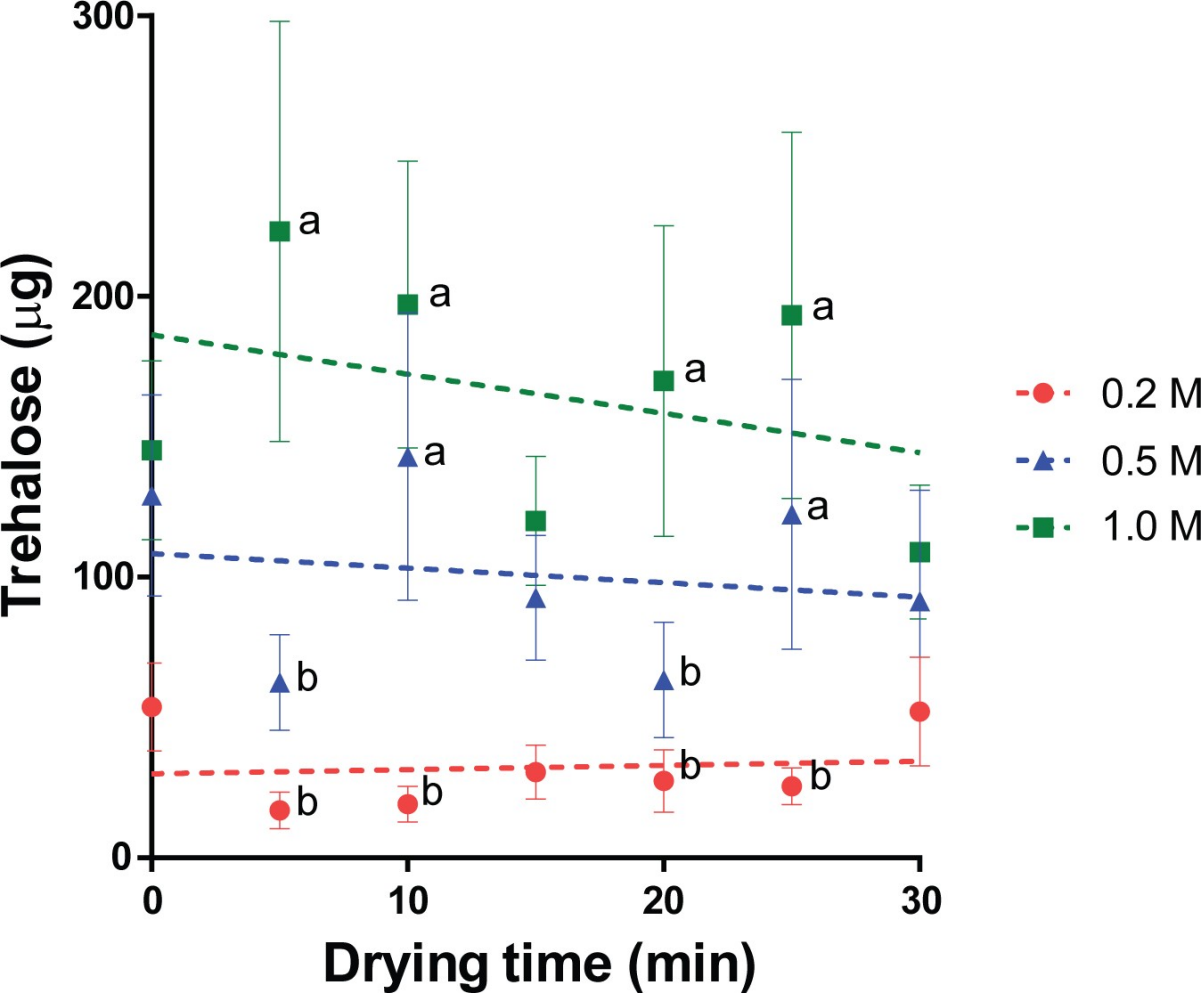
Trehalose content in tissues during microwave drying. Values are mean ± SEM. Two-way ANOVA analysis indicated significant effect (*P* < 0.05) of trehalose concentration, but not drying time, on trehalose content. Samples treated with same concentration of trehalose exhibited no significant differences (*P* > 0.05) over different drying time. At each time point, values with different letters differ (*P* < 0.05) among groups exposed to different concentrations of trehalose at the same drying time.

Trehalose was detectable in all samples that were dried within 30 min, indicating that trehalose remained within the tissues during water loss. At given time points (5, 10, 20 and 25 min), trehalose contents in tissues were consistently higher (*P* < 0.05) when samples were initially exposed to higher trehalose concentrations (0.5 or 1 M; Fig 3). However, there were no differences across drying times (P > 0.05) among samples exposed to the same initial concentration of trehalose (range, 0.2 M: 16.9 to 53.8 μg, 0.5M: 62.5 to 143.0 μg, and 1.0 M: 108.9 to 223.2 μg per piece; Fig 3). Importantly, we verified that trehalose could be detected only after cell membrane disruption by freezing/thawing (S2 Fig). This indicated that the protectant had indeed penetrated the cells in the tissue biopsies.

**Fig 4:**
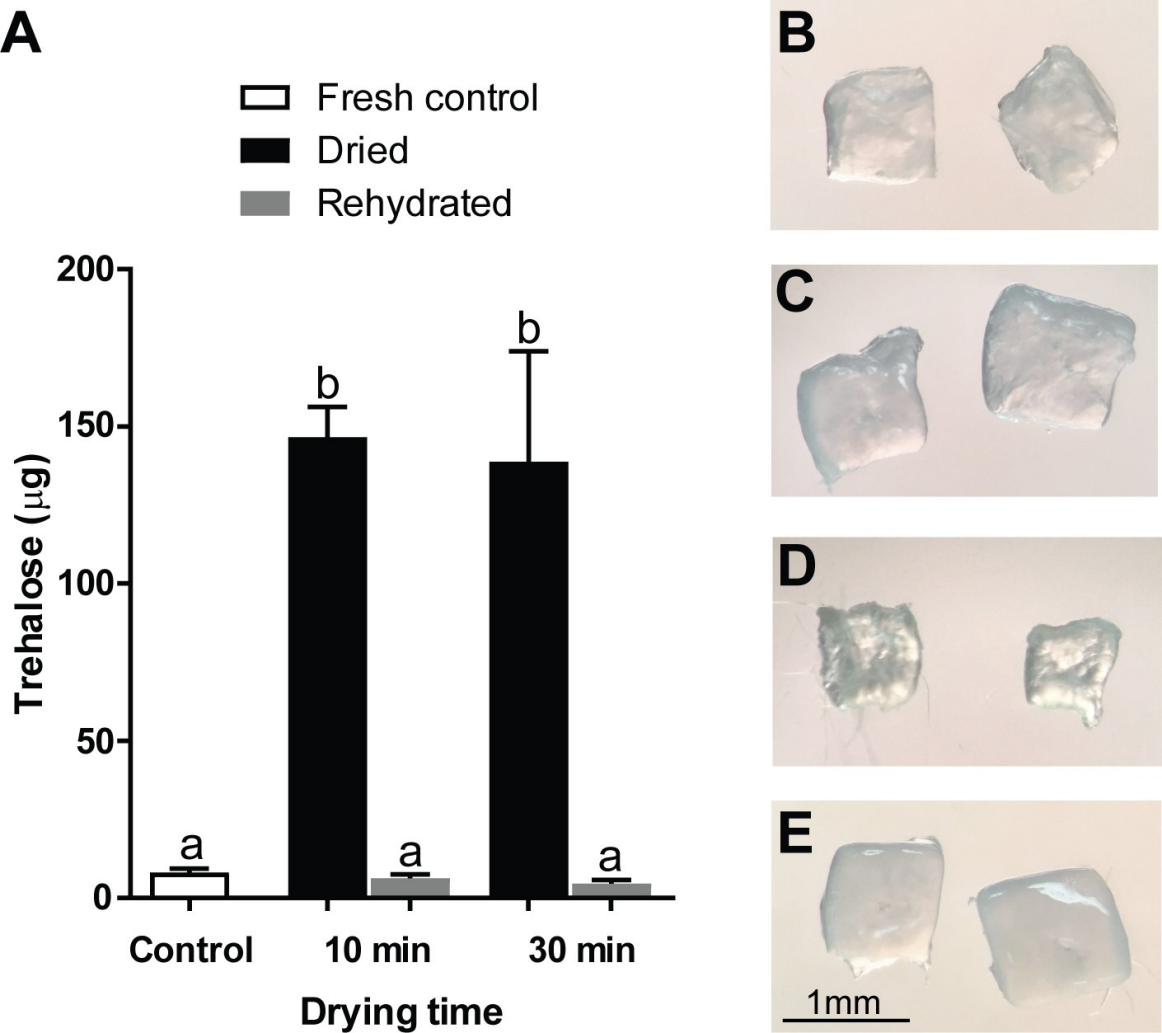
Effect of drying and rehydration on trehalose content and gross morphology of ovarian tissue. (A) Trehalose levels after dehydration and rehydration. Values are mean ± SEM. Values with different letters differ (*P* < 0.05). (B-E) Gross morphology of whole ovarian cortical pieces that were (B) un-treated, (C) exposed to 0.5 M trehalose for 10 min, (D) dried for 30 min, and (E) rehydrated for 30 min.

The goal of preservation is to be able to return biomaterials to the same state prior to drying, therefore trehalose loading must be reversible upon reanimation. To verify this, effect of rehydration after trehalose exposure and microwave drying was further analyzed using 0.5 M trehalose as a reference. As previously reported, trehalose contents were maintained in tissues after 10 or 30 min of drying (range, 138.8 to 146.6 μg per piece; Fig 4A). After 30 min of rehydration, trehalose content returned to a background level regardless of degree of dehydration (Fig 4A). Moreover, gross morphology of cortical pieces did not change after trehalose exposure compared to the fresh controls (Fig 4B and 4C). Although shrinkage and hardening of cortical pieces was apparent after microwave dehydration (Fig 4D), gross appearance of tissues returned to normal after rehydration (Fig 4E).

### Effect of drying time on tissue histology, DNA integrity, and transcriptional activity

**Fig 5:**
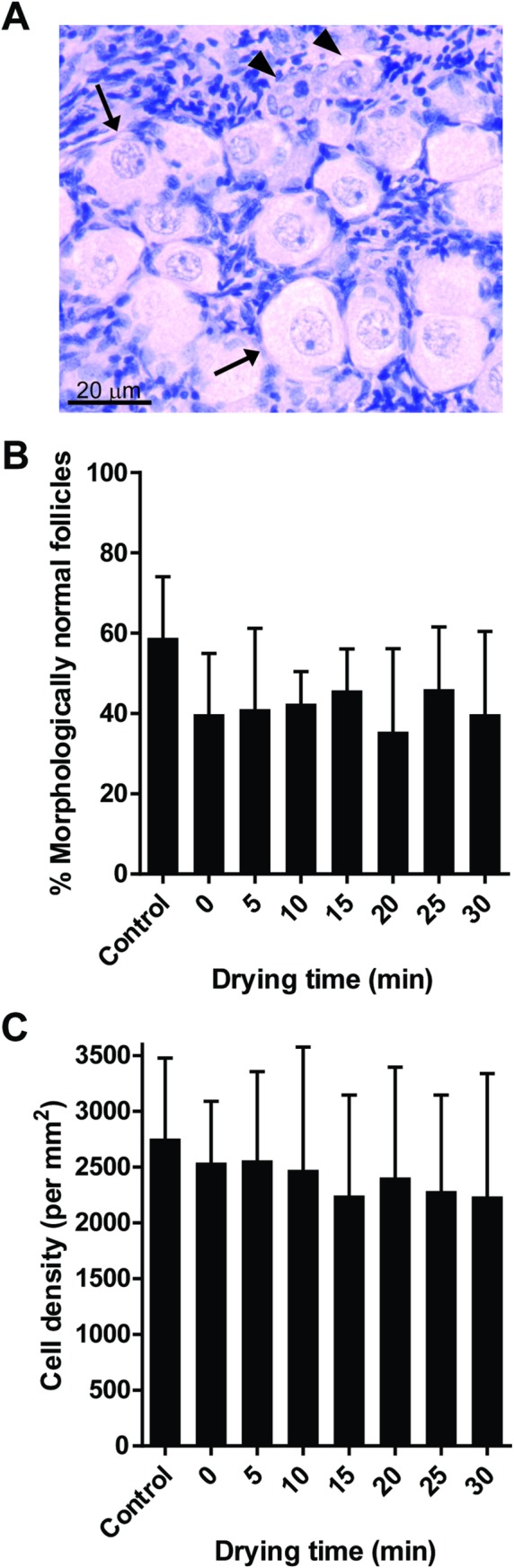
Effect of drying time on ovarian tissue histology. (A) Representation of morphologically normal (arrows) and abnormal (arrowheads) follicles after fixation and haematoxylin–eosin staining. (B-C) Analyses of (B) follicular morphology and (C) stromal cell density of tissue pieces dried for up to 30 min. Values are mean ± SD. No statistically significant (*P* > 0.05) differences were observed.

**Fig 6:**
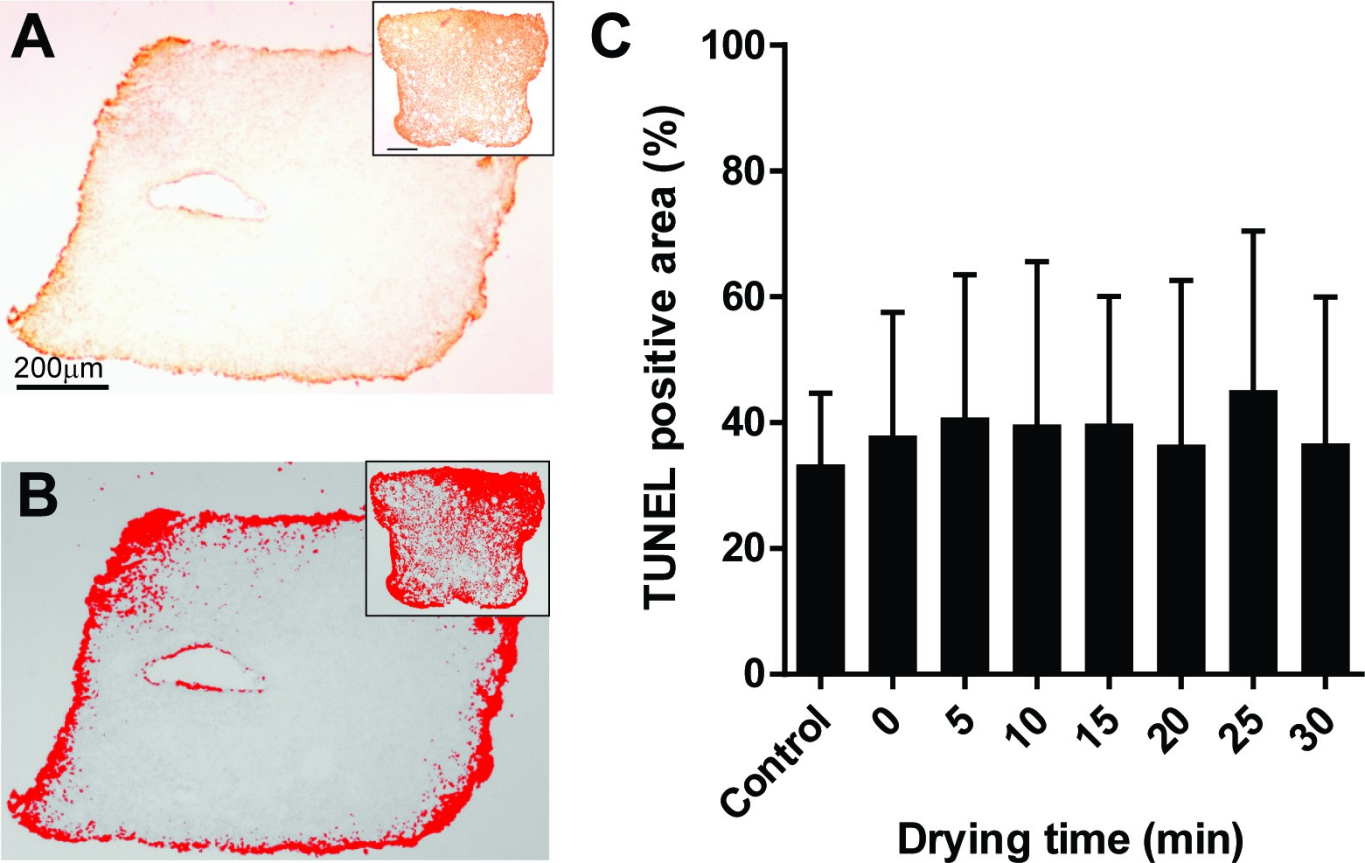
Effect of drying time on DNA integrity. (A) Representation of TUNEL-positive staining (brown) indicating DNA damage in the cortical piece. Inset: DNase-treated positive control tissue. Scale bars = 200 μm. (B) Quantification of TUNEL-positive area using ImageJ. Inset: DNase-treated positive control tissue. (C) Analysis of DNA integrity of tissue pieces dried for up to 30 min. Values are mean ± SD. No statistically significant (*P* > 0.05) differences were observed.

**Fig 7:**
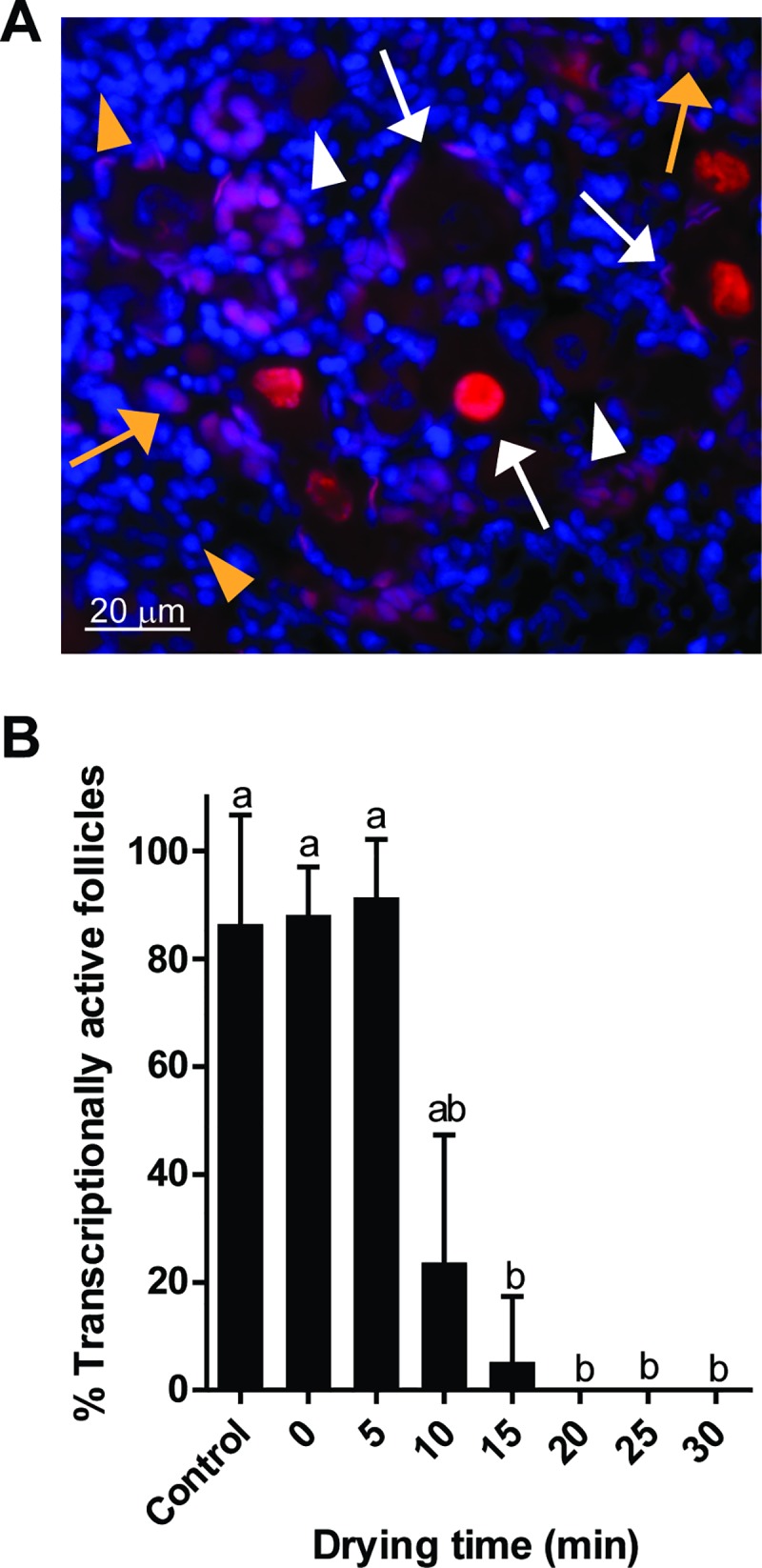
Effect of drying time on transcriptional activity. (A) Representation of transcriptional activities after EU fluorescent labelling. White arrows: transcriptionally active follicles, white arrowheads: transcriptionally inactive follicles, yellow arrows: transcriptionally active somatic cells, and yellow arrowheads: transcriptionally inactive somatic cells. (B) Analysis of follicular transcriptional activity in tissue pieces dried for up to 30 min. Values are mean ± SD. Values with different letters differ (*P* < 0.05).

Once effective drying and rehydration were validated, we assessed follicular integrity to characterize the impact of drying level on the tissue. Gross appearance of dried/rehydrated tissue sections, even up to 30 min of drying, showed no distinguishable shrinkage, breakage, or disintegration other than the hole created by needle threading during tissue manipulation. Histological analysis showed that percentages of morphologically normal follicles tended to slightly decreased in trehalose-exposed tissue compared to fresh controls (58.9%) but were not affected (*P* > 0.05) by drying times (range, 35.5 to 46.1%, Fig 5A and 5B). Similarly, stromal cell density remained unchanged (*P* > 0.05) during drying (range, 2236 to 2756 cells/mm^2^, Fig 5C). Terminal deoxynucleotidyl transferase dUTP nick end labeling (TUNEL) revealed that most of the cells with DNA damage were located at the rim of the tissue pieces or around the needle-pierced hole (Fig 6A and 6B). The proportion of TUNEL-positive region in the control tissue pieces was 36.6%. Microwave-drying, from 0 to 30 min, did not significantly increase the proportion of TUNEL-positive region (range, 39.5 to 47.7%, Fig 6C). Post-rehydration transcriptional activity was maintained (range, 88.1 to 91.4%) through 5 min of desiccation (Fig 7A and 7B). However, decrease in percentages of transcriptionally active follicles was observed after 10 min of drying (23.7%, *P* > 0.05) and was more significant after 15 min (5.3%, *P* < 0.05). Transcriptional activity was not detected after 20 min of drying and rehydration (Fig 7B). Similarly, transcriptional activity in the somatic cells was partially retained after 10 and 15 min of drying, and none observed by 20 min (Fig 7A).

### Effect of short-term storage and trehalose concentrations on tissue histology and transcriptional activity

**Fig 8:**
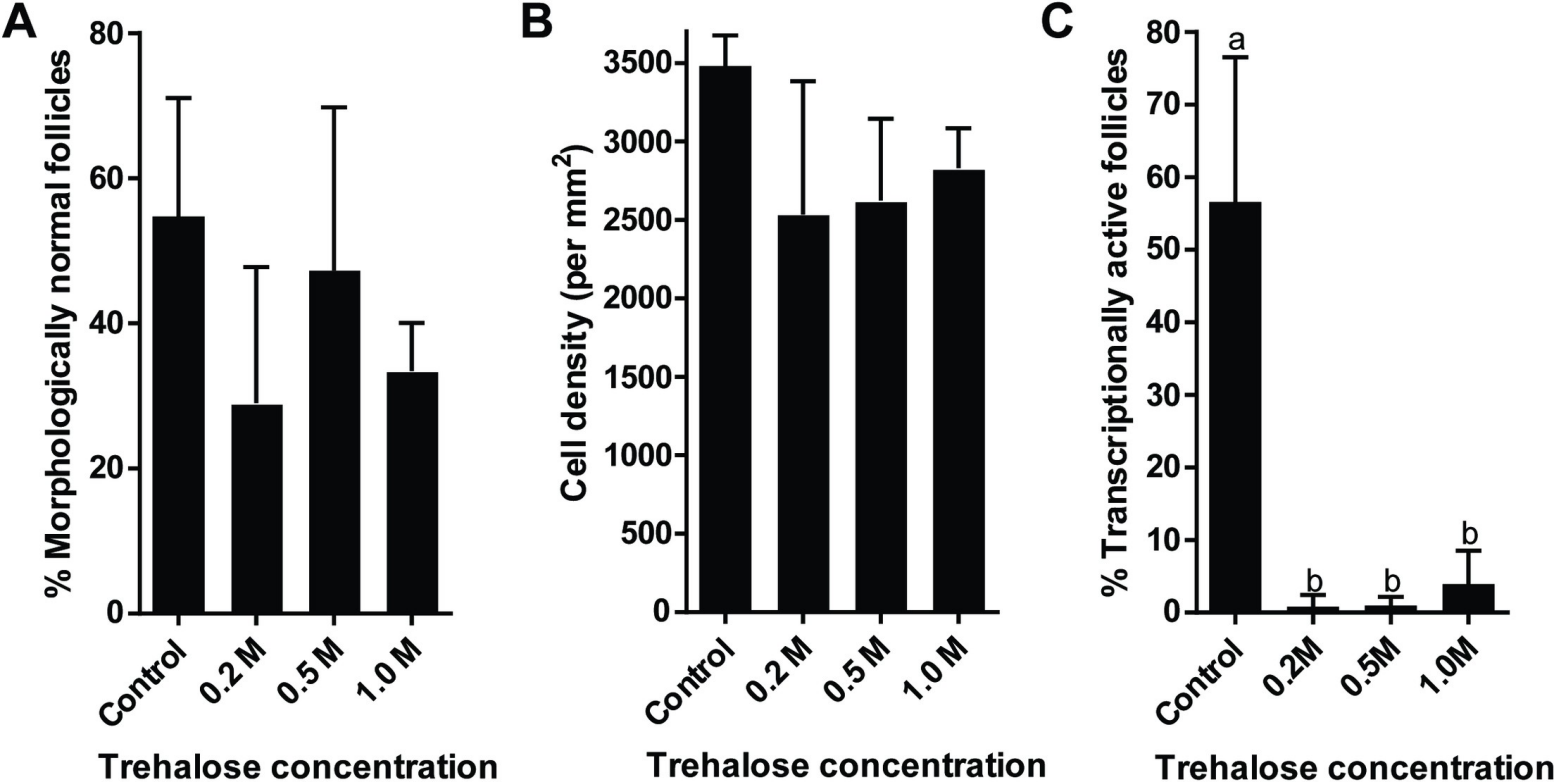
**Effect of different trehalose concentrations on (A) follicular morphology, (B) stromal cell density, and (C) transcriptional activity after 1d storage at 4°C.** Values are mean ± SD. Values with different letters differ (*P* < 0.05). No statistically significant (*P* > 0.05) differences were observed in analyses for follicular morphology and cell density.

Based on the previous experiments, 10 min of drying produced the best outcome on tissue integrity while reaching a reasonably low water content. Using the same drying time, we then examined the effect of short-term storage at 4°C and trehalose concentrations (0.2, 0.5, or 1.0 M) on ovarian tissues. No statistical difference (*P* > 0.05) was observed in percentages of morphologically normal follicles among ovarian tissues exposed to any of the three trehalose concentrations (range, 29.0 to 47.3%) or the control group (54.8%, Fig 8A). Stromal cell density tended to decrease but remained stable (*P* > 0.05) in dried-stored groups (range, 2535 to 2831 cells/mm^2^), which was similar to the control group (3481 cells/mm^2^, Fig 8B). Compared to fresh controls (58.3%), proportion of transcriptionally active follicles was diminished (*P* < 0.05) to less than 1.0% in 0.2 M and 0.5 M trehalose-exposed biopsies (Fig 8C). While not significantly higher (*P* > 0.05) than the levels in 0.2 and 0.5 M trehalose-exposed tissues, 4.0% of follicles remained transcriptionally active when tissues were exposed to 1.0 M trehalose (Fig 8C). Although improvements are necessary, the small percentages of transcriptionally active follicles, especially in the 1M trehalose-treated group, hinted the possibility to sustain viability after drying and storage at supra-zero temperatures.

### Effect of drying time and storage on tissue survival

**Fig 9:**
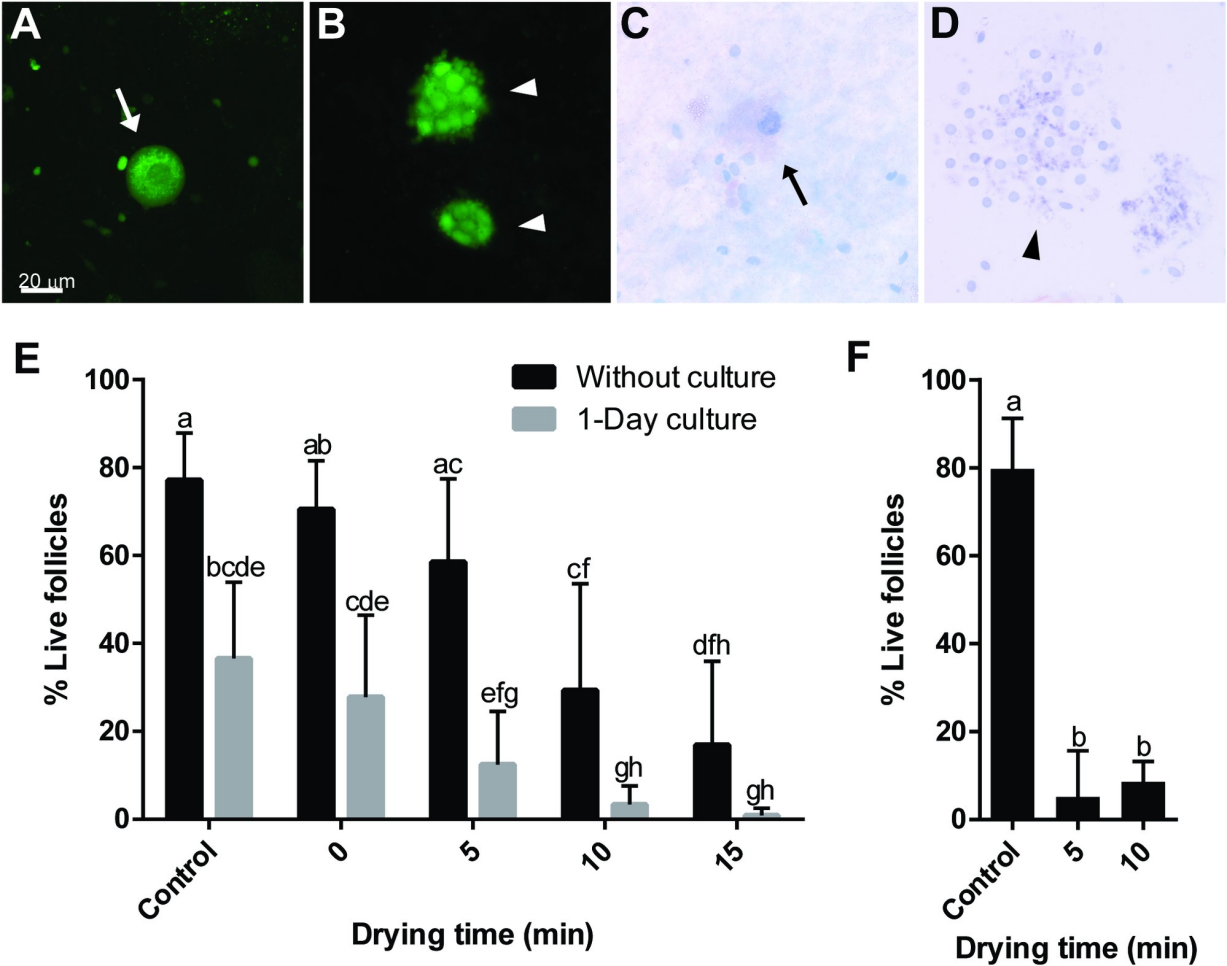
Effect of drying time and storage on follicle survival. (A-D) Representation of live and dead follicles after Calcein AM and trypan blue staining, respectively. (A) Live oocyte (arrow); (B) live granulosa cells (arrowheads); (C) dead oocyte (arrow); (D) dead granulosa cells (arrowhead). (E) Analysis of follicle survival in fresh control tissue and tissue pieces dried for up to 15 min and either assessed immediately after rehydration or after 1-day culture. (F) Analysis of follicle survival in fresh control tissue and tissue pieces dried for 5 or 10 min and storage for 24 h. Values are mean ± SD. Values with different letters differ (*P* < 0.05).

After exploring the effect of drying time and storage on tissue histology, DNA integrity and transcriptional activity, we assessed how these collective phenotypes translated into overall tissue survival (Fig 9A–9D). Most of the follicles survived after 5 min of drying. Although a significant decrease (*P* < 0.05) was observed after 10 and 15 min of drying, there were still 29.3% and 16.9% of live follicles in the tissue cortex, respectively (Fig 9E). After 1-day culture, about half of the follicles in the fresh tissue survived. Results from 1-day cultured tissue showed a similar trend as non-cultured counterparts with overall lower percentages of live follicles: 12.4% with 5 min, 3.4% with 10 min and 0.9% with 15 min of drying (Fig 9E). We further observed that proportions of live follicles significantly decreased (*P* < 0.05) to 5.2% in samples with 5-min drying and 1 day storage, compared to fresh control (79.7%, Fig 9F). The proportion was slightly higher when tissues were dehydrated for 10 min (8.6%) instead of 5 min, yet the difference was not statistically significant (*P* > 0.05). It is noteworthy that almost all follicles died after 1 day storage when tissues were dried to a comparable water content (20 min drying) in the absence of trehalose (S3 Fig). Therefore, trehalose was essential to protect tissues from desiccation damages during dry-storage.

## Discussion

The present study explored for the first time the use of microwave-assisted dehydration to preserve living ovarian tissues. Biopsies of ovarian cortex could be efficiently dehydrated while retaining exogenous trehalose to stabilize biological functions. Specifically, follicular morphology, DNA integrity, and partial viability were maintained in tissues after drying followed by storage at a supra-zero temperature and rehydration. Collective results demonstrated that dry-preservation of living mammalian tissue samples is possible although further studies are required to refine those approaches inspired from desiccation-tolerant organisms.

Microwave provides a fast and uniform way to remove water molecules from samples. Understanding the drying kinetics is an important first step toward dry-preservation of biomaterials. When analyzing protectant solutions, water content is commonly expressed as gH_2_O/gDW. In previous reports on single cell suspensions, weight of biomaterials was neglected in water content calculations [28, 29]. In the present study, measurement of dried solute weight would have been masked by the substantial weight of tissues. We therefore expressed water content in two different ways—water weight and percentage of water in each piece of tissue. Resulting drying curves resembled those observed with single cells, albeit use of different units [28, 29]. Kinetics was also similar to what was observed in microwave drying of food products, with an initial rapid drying phase followed by a slow drying period with gradually falling drying rate until equilibrium is reached [33, 34]. Several studies in drying of vegetables and fruits, which also are large and possess heterogeneous composition, have determined that the logarithmic model is best fitted model for their drying curves [35–37]. A similar model was also suitable for our drying data with high degrees of fitness. In particular, R^2^ for logarithmic fitting of water percentage data were higher than those for water weight. Hence, percentage of water would be a better format for the drying kinetic data for using logarithmic model to predict water content in living tissues. It is noteworthy that fluctuated relative humidity and room temperature in current experimental conditions likely affected the drying kinetics of samples processed on different dates, which in turn led to high variation in the outcome of tissue integrity analyses. We are currently establishing a working station with controlled environment, which would limit the variations.

Trehalose is generally considered as a non-toxic agent for preservation of living cells [23]. Critical to the success of dry-preservation, exogenous trehalose needs to enter and remain in the cellular compartments during desiccation to improve desiccation tolerance of cells. We demonstrated here that trehalose was retained within ovarian tissue during the course of microwave drying. Importantly, trehalose could only be released and detected after membrane disruption, indicating that trehalose had reached the intracellular compartments. Evidence from our pilot study also indicated the beneficial effect of digitonin to induce membrane permeabilization that enabled penetration of trehalose into the tissue. While many studies have focused on ensuring and improving trehalose uptake, very little is known about the unloading process during reanimation. Here we reported that trehalose levels within tissue biopsies returned to background levels upon rehydration, even after prolonged drying, suggesting that trehalose loading was fully reversible. This could be advantageous over cryopreservation in which residual cryoprotectants have been reported after warming/thawing of ovarian tissues [38]. Although trehalose was detectable within all the trehalose-exposed tissues, the amount widely varied even among the ones that underwent the same treatment. While minor size differences among tissue pieces may account for part of the observed variation, trehalose delivery methods could also affect the outcome. In the current protocol, trehalose loading relied on passive diffusion through digitonin-permeabilized membrane. Other methods, such as trehalose transport via polymers [39, 40] or nanoparticles [41, 42], have been proven effective for loading of high amount of trehalose and should be examined in the future for more consistent intracellular delivery of trehalose.

Our results suggested that tissue morphology was tolerant of dehydration stress during microwave-assisted drying. Other than a mild decrease on the proportion of morphologically normal follicles upon initial trehalose exposure, which was likely due to the hyperosmotic effect of trehalose solution, microwave drying did not lead to additional morphological abnormalities. Interestingly, this is comparable to the data obtained from previous cryopreservation studies on cat ovarian tissue, suggesting that follicular structure could be stabilized under both isothermic and cryogenic vitrification procedures [11, 43].

Investigation by Baust et al. revealed that without protective agents, desiccation induced apoptosis in mammalian cells through activation of caspase cascade [44]. Previous studies on microwave-dried germinal vesicles and spermatozoa using trehalose as the protectant showed resilience of DNA to desiccation stress [28, 29]. In the presence of trehalose, TUNEL analysis in this study also showed no significant difference between fresh control and dried/rehydrated tissue. Moreover, stromal cell density remained the same in dried/rehydrated tissue, suggesting that no significant cell degeneration has occurred [45]. However, significant decrease in live follicles was observed in tissues dried for over 10 min. Taken together, current microwave-assisted dehydration protocols do not induce additional DNA damage yet adversely affect follicle survival with longer drying time.

Despite relatively unaltered morphology, transcriptional activity was negatively affected by dehydration. Transcriptional activities completely ceased in both rehydrated follicles and stromal cells after 20 min of drying, corresponding to a water content of 7.2% or lower. This is consistent with previous notion that drier state does not necessarily produce better preservation outcomes [28, 46]. New evidence suggests that trehalose protects proteins by forming a shell that traps water around these macromolecules [47]. However, over-drying could deteriorate the dynamic among water molecules, trehalose matrix, and intracellular macromolecules and adversely affect cell function and viability [20, 47]. The drying level reached at 20 min may have dropped below the threshold of water content critical for cellular functions in the ovarian tissue [20]. Encouragingly, transcriptionally active follicles and stromal cells were observed, although with low frequency, after 10 and 15 min of drying, corresponding to 11.6% and 7.2% of water in the tissue, respectively. This suggests that basic functionality could potentially be preserved in some cells in the ovarian tissue. Noticeably, decreased transcriptional activity in the follicles has also been reported in the vitrified cat tissue, although to a lesser extent than what was observed in the dried counterparts [11]. These data suggest that RNA transcription is a more sensitive indicator than histological analysis to reflect the influence of external stress, such as vitrification and desiccation, on the vital functions of ovarian tissue.

Successful synthesis of RNA transcripts requires intact DNA templates as well as a combination of proteins that make up the transcription machinery. The observed loss of transcriptional activity within dehydrated ovarian tissue was not caused by increase of DNA fragmentation as revealed by TUNEL assays. We suspect that proteins involved in transcription might have been destabilized and lost their function. It is known that desiccation may cause protein denaturation, degradation, or aggregation [19, 48]. If this hypothesis is correct, it is likely that other protein-dependent cellular functions would also be influenced by dehydration. Although trehalose possesses the ability to protect proteins against these detrimental effects [19, 48], over-drying and uneven distribution of trehalose in complex biomaterials may have impaired the biostabilization property.

The most attractive potential for dry-preservation is the possibility to store biomaterials at supra-zero temperatures, and hence avoid handling and management of liquid nitrogen or freezers. Three different trehalose concentrations were tested in the storage experiment. The differences in concentration could influence the tissue in two manners. First, these solutions possess different osmolarities, which likely result in differential impact on tissue morphology and stress response upon exposure as well as varied distribution of trehalose within the tissue [49, 50]. Second, water contents differ after drying when exposed to different concentrations of trehalose according to the drying curves, thereby provide a range of theoretical T_g_ for the storage study. When examining dried/rehydrated ovarian tissue after being stored at 4°C, both proportion of morphologically normal follicles and stromal cell density tended to decrease, although not significantly different from control samples, regardless of trehalose concentrations. This suggest that cell degeneration was limited during storage. However, the results also showed that transcriptional activity was further compromised after storage. Only 4% of the follicles in 1 M trehalose-exposed tissue were active, and less than 1% in 0.2 and 0.5 M treatment groups. This indicated that cellular environment was not stable during storage at any of the water contents provided. A recent study reported similar results on freeze-dried fibroblasts that DNA damage progressively increased during storage at supra-zero temperatures [51]. On the other hand, it has been demonstrated that microwave-dried oocytes maintained DNA integrity when stored at 4°C or ambient temperature for up to 8 weeks [28]. The source of the discrepancy may be the different T_g_ achieved in each study. In our case, it is likely that the actual T_g_ within ovarian tissue was lower than the theoretical value. More research is required to improve desiccation tolerance in tissues to allow preservation at drier condition (and hence higher temperature).

Lastly, we demonstrated that follicle survival was at least partially maintained with up to 15-min of drying. This is consistent with our results on transcriptional activities. Both assays rely on enzymatic activities: RNA polymerases for transcription and esterases for Calcein AM live cell staining, and could be used as indicators for follicular viability. We therefore concluded that at least some functional enzymes could be protected in the follicles when desiccated. In this experiment, dead follicles were determined by the ability of trypan blue dye to enter compromised membrane. It is worth noting that the proportion of dead follicles in digitonin-treated, non-dried (0 min drying) tissue was similar to fresh control samples (Fig 9E). This suggests that membrane damage observed in dried tissues was not resulted from digitonin exposure, but rather from the desiccation/rehydration process. Furthermore, effect of stress exposure can sometimes be delayed or mitigated in tissue culture [15]. While the culture system remains to be improved, no noticeable mitigation was observed on dry-preserved tissue under current in vitro culture protocol. Importantly, some rehydrated follicles remained viable in tissue dried for 10 min after 24 h culture. This indicates that follicles possess the capability to withstand desiccation stress, and our data provide the foundation for future optimization of the technique. Although follicle survival was fully preserved immediately after 5 min of drying (Fig 9E), proportion of live follicles greatly decreased after storage at 4°C (Fig 9F). This was anticipated because water content within the tissue was too high to allow glass transition of trehalose at higher temperatures. The decline in viable follicles was less drastic when comparing 10-min dried tissues before and after storage (Fig 9E and 9F). Consistent with our assessment on transcriptional activity, small proportion of follicles remained alive. The results support our earlier conclusion that it is possible to sustain follicular viability during storage at supra-zero temperatures; however, it will be necessary to reach a lower water content in order to increase biostability.

This study is the first exploration of dry-preservation of the ovarian tissue. Under the current protocol, 1 M trehalose exposure and 10 min of microwave drying yielded the best balance between desired low water content and partial maintenance of follicular viability. Multiple steps within the current procedure, including trehalose loading method, xeroprotectant composition and exposure, microwave setting, and storage condition, will have to be adjusted. However, present data are paving the way for new preservation approaches that will change the paradigm in biopreservation and biobanking.

## Materials and methods

### Collection of ovarian tissues

All methods were carried out in accordance with relevant guidelines and regulations. The study did not require the approval of the Animal Care and Use Committee of the Smithsonian Conservation Biology Institute because cat ovaries were collected at local veterinary clinics as byproducts from owner-requested routine ovariohysterectomies. Ovaries from peri-pubertal domestic cats were recovered and transported at 4°C to the laboratory within 6–18 hours of excision. Ovarian cortical tissues were dissected into 1 x 1 x 0.2 mm pieces in dissection medium (minimum essential medium [MEM with Hank’s salt; Gibco Laboratories] supplemented with 10mM HEPES, 1 mM pyruvate, 2 mM L-glutamine, 100 IU/ml penicillin, 100 μg/ml streptomycin and 0.1% bovine serum albumin [BSA]) [11]. All chemicals are from Sigma-Aldrich unless otherwise indicated. Cortical pieces from the ovaries of a single cat was considered as one replicate.

### Microwave-assisted dehydration, storage, and rehydration

Relative humidity was 6.9 to 71.2% and room temperature was 21.2 to 28.4°C during the course of the study. All procedures were carried out at room temperature unless otherwise indicated. A needle immersion technique was adapted for ease of manipulation [11]. Cortical pieces were threaded onto a 30-G needle (2–3 pieces per needle; Fisher Scientific) with space in between. Microwave drying procedure was modified from previously described protocol [28]. Cortical pieces were immersed in 10 ug/ml digitonin for 3 min to permeabilize the tissue. After rinsing in dissection medium, cortical tissues were exposed to trehalose for 10 min. Excess trehalose solution was gently dabbed off on kimwipes before moving the cortical pieces from the needle onto conjugate-release glass fiber filters (Whatman). Samples were dehydrated in a SAM 255 microwave (CEM) for the desired length of time at 20% power (about 100 W power output) with upper temperature threshold set at 40°C. For storage, filters were individually sealed in Dri-shield moisture barrier bags (3M) and stored at 4°C. For rehydration, filters were immersed in dissection medium for at least 30 min at room temperature, and then rehydrated cortical pieces were collected for analyses.

### Assessment of water content in tissues

Two pieces of cortical tissues were used for each water content measurement. The total weight (filter weight excluded) of the pair of tissues was measured with analytical balance (XP-150, Mettler Toledo) immediately before samples underwent microwave dehydration. Water weight of tissue pieces was measured with a Karl Fisher titrator (Mettler-Toledo V20) [28]. Water content was presented as water weight per tissue piece and as percentage of water weight relative to total tissue weight per tissue piece. Drying curves were obtained by fitting data into a logarithmic model using non-linear regression (GraphPad Prism 6; GraphPad Software) [35–37].

### Assessment of trehalose content in tissues

Ovarian tissue pieces underwent indicated microwave drying procedure, excess trehalose was dabbed off from the tissues with kimwipes. Tissues were snap frozen and kept in -80°C until all samples were ready for analysis, then thawed at the same time to disrupt cell membrane before trehalose extraction. Each piece was placed in a single well of a 96-well plate and trehalose was extracted by adding 200 μl of hot (80°C) distilled water to each well and shaking at 600 rpm for 15 min. Each sample was diluted 1:10 and trehalose content was quantified with a trehalose assay kit (Megazyme) following manufacturer’s instruction. The kit utilized a series of enzymatic reactions to convert trehalose into reduced nicotinamide-adenine dinucleotide phosphate, which could be measured by the absorbance at 340 nm with a BioTek ELx808 microplate reader (BioTek Instruments).

### Assessment of tissue and follicular morphology

Follicular morphology was assessed via haematoxylin–eosin staining. Cortical pieces were fixed in Bouin’s solution, embedded in paraffin, and sectioned at a thickness of 5 μm. Haematoxylin–eosin staining was performed on 3 non-consecutive sections with 30 μm intervals to avoid double-counting of follicles. Both primordial (oocytes with single layer of flattened granulosa cells) and primary follicles (oocytes with single layer of cuboidal granulosa cells) were included for analysis. Follicles were classified as abnormal when exhibited any of the following characteristics: detachment of basement membrane, pyknotic nuclei, oocyte cytoplasmic vacuolization, and granulosa cell disruption. Otherwise, the follicles were classified as morphologically normal. Stromal cell density was analyzed with ImageJ (NIH). Briefly, two section areas from each treatment group were randomly selected. Haematoxylin staining was isolated via colour deconvolution and converted to binary image via threshold adjustment, and numbers of nuclei were determined by particle analysis.

### Assessment of DNA integrity

To detect DNA damage in the ovarian tissue, cortical pieces were assayed with the Click-iT TUNEL colorimetric IHC detection kit (Life Technologies) following the manufacturer’s instruction. Tissue sections were prepared as described above. In brief, slides were deparaffinized, permeabilized with proteinase K, and then underwent TdT reaction to incorporate EdUTP (an alkyne-modified dUTP) to the ends of fragmented DNA. After quenching and sufficient washing, slides were exposed to Click-iT colorimetric reaction cocktail containing biotin azide and then to streptavidin-peroxidase conjugate. Staining color was developed by DAB reaction. A positive control in which tissue sections were exposed to DNase before TdT reaction and a negative control in which TdT enzyme was omitted were included in each replicate. Slides from all treatments from the same replicate were process simultaneously to allow comparison. The percentage of TUNEL positive area was measured with ImageJ using threshold levels determined through positive and negative controls [52].

### Assessment of transcriptional activity

To determine transcriptional activity in the tissues, nascent RNAs were labeled and imaged with the Click-iT RNA imaging kit (Life Technologies). Fresh or rehydrated cortical pieces were incubated with 1 mg/ml 5-ethynyl uridine (5-EU) in culture medium consisting of MEM (with Earle’s salts; Sigma-Aldrich) supplemented with 2 mM L-glutamine, 100 UI/ml penicillin, 100 μg/ml streptomycin, 50 μM ascorbic acid, 10 μg/ml insulin, 5.5 μg/ml transferrin, 6.7 ng/ml selenium (ITS; Gibco Laboratories), 0.3% poly(vinyl) alcohol and 10 μg/ml follicle stimulating hormone (Bioniche Animal Health). A negative control without 5-EU incorporation was included in each replicate. After 4 h incubation, tissue pieces were fixed in 4% paraformaldehyde. Fixed samples were embedded and sectioned as previously described. 5-EU was detected following the manufacturer’s instruction. In brief, after paraffin removal and rehydration, tissue sections were washed in 3% BSA in Dulbecco’s phosphate buffered saline (DPBS), permeabilized with 0.5% Triton X-100/DPBS for 30 min, rinse in 3% BSA/DPBS, and then exposed to the Click-iT reaction cocktail containing Alexa Fluor 594 azide. After rinsing, each slide was counterstained with Hoechst 33342 and mounted with Vectashield mounting medium (Vector Laboratories). Fluorescent signals were visualized with the Olympus BX41 epifluorescence microscope. Follicles were classified as transcriptional active if oocyte and/or at least one surrounding granulosa cell show positive Alexa Fluor 594 staining.

### Assessment of follicle survival

Two cortical pieces per group were used to assess follicle survival in fresh or rehydrated tissues. Follicle survival was determined using a cell-permeant dye Calcein AM (Invitrogen) to stain live intact cells and a non-permeating dye trypan blue (Sigma-Aldrich) to stain dead or damaged cells. To better visualize individual follicles during the assay, cortical pieces were first lightly digested by incubating them in DPBS with 0.08 mg/ml Liberase^™^ DH (Roche Life Scientific) and 5% FBS at 38.5°C for 30 min, mixing by hand every 5 min to disrupt the tissue. The digestion was stopped by adding an equal volume of 10% FBS in cold DPBS, and the softened cortical pieces were transferred to 2 μl/ml Calcein AM in DPBS and incubated at 38.5°C for 30 min. The cortical pieces were then transferred to 1:50 ProLong Live Antifade Reagent (Invitrogen) in DPBS for the duration of analysis and kept at room temperature. One by one, each sample was first incubated in 0.2% trypan blue in DPBS for 3 min at room temperature, then transferred to a microscope slide, with cover slip gently pressed down on the tissue to obtain a squash preparation and analyzed immediately. The sample was visualized at 518 nm first to count all live follicles, and then using the bright-field to count all dead follicles. Follicles were considered alive when the oocyte and surrounding granulosa cells fluoresced green, and were considered dead when the oocyte and/or at least 50% of the surrounding granulosa cells stained blue. Proportions of live follicles were calculated by dividing the number of live follicles by the sum of live and dead follicles.

### In vitro culture of ovarian tissue pieces

Ovarian cortical tissue pieces were cultured according to Fujihara et al [53]. Briefly, 1.5% (w/v) agarose gel was cut into 1 x 1 x 1 cm3 inserts and pre-conditioned in culture medium consisting of Eagle MEM with Earle’s balanced salts supplemented with 2mM L-glutamine, 100 UI/ml penicillin, 100 μg/ml streptomycin, 50μM ascorbic acid, 10 μg/ml insulin, 5.5 μg/ml transferrin, 6.7 ng/ml selenium (the last three as ITS+; Gibco Laboratories), 0.3% poly(vinyl) alcohol and 0.025 AU/ml porcine follicle stimulating hormone (Sigma-Aldrich). The cortical pieces were cultured for 24 h on the agarose gel inserts (4 pieces/insert) in 4-well culture dishes (Thermo Scientific) with 400 μl of culture medium at 38.5°C in 5% CO_2_ in air.

### Experimental design

Our general experimental design was modified from microwave-assisted dehydration protocols previously developed. Digitonin was selected as the agent to reversibly permeabilize cellular membranes [54] and allow transient intracellular penetration of trehalose. A pilot study confirmed that tissues permeabilized with digitonin contained a higher percentage (*P* < 0.05) of morphologically normal follicles after drying than controls without digitonin treatment (S1 Fig).

First, we explored the kinetics of water loss and trehalose retention in ovarian tissue during microwave drying. Digitonin-treated cortical tissues were exposed to one of the four trehalose concentrations tested (0, 0.2, 0.5 or 1.0 M). Water content was measured for every 5 min interval of processing, up to 40 min (n = 54, in 6 replicates for each concentration). Similarly, kinetics of trehalose content in biopsies was measured after dehydration at different time points for up to 30 min (n = 126, in 6 replicates; Fig 1). Additionally, we examined the effect of rehydration on trehalose content and gross morphology of ovarian tissue. We chose 0.5 M trehalose as a reference for this experiment. Trehalose-exposed cortical tissues were dried for 10 or 30 min. Tissues were processed for trehalose measurement either immediately after drying or after 30-min rehydration (n = 30, in 6 replicates; Fig 1). Gross morphology of the tissue pieces was observed using a stereo microscope after tissue dissection, digitonin and trehalose exposure, drying, or rehydration.

Next, we assessed the effect of drying time on tissue integrity. As proceeded before, the starting trehalose concentration of 0.5 M was used as a reference. A pair of fresh tissue pieces from each replicate was fixed without any treatment to serve as control. Rest of the pieces underwent microwave dehydration procedure for up to 30 min. These cortical pieces (including 0 min-microwaved samples) were rehydrated immediately for 30 min before histological evaluation, TUNEL assay, and transcription assay (n = 48, in 6 replicates; Fig 1).

Results of the last experiment showed that 10 min of microwaving allowed maintenance of morphology and transcriptional activity in a portion of follicles and somatic cells under a low water content of 11.6%. According to the Gordon-Taylor equation [32], T_g_ at this water content is 4.7°C in pure trehalose solution, which in theory supports storage at 4°C in refrigerators. However, this calculation may not be directly applicable to complex biomaterials with significant mass. We therefore examined the effect of storage using initial tissue exposures to 3 different concentrations of trehalose, 0.2, 0.5 and 1.0 M. Ovarian tissue pieces were exposed to one of the three trehalose concentrations, microwave dried for 10 min, and stored at 4°C in moisture-barrier bags for at least 24 hours before rehydration and evaluation (n = 20, in 4 replicates; Fig 1).

Lastly, we assessed the effect of drying time and storage on tissue survival. In the first part of the experiment, ovarian tissue pieces were microwave dried for 0, 5, 10, or 15 min after exposed to 1 M trehalose, and rehydrated right after. Half of the tissue pieces were assayed for follicle survival immediately, and the other half were cultured for 24 h before evaluation (n = 70, in 7 replicates; Fig 1). Fresh, untreated biopsies were included as controls. In the second part of the experiment, tissue pieces were dehydrated for 5 or 10 min, stored at 4°C in moisture-barrier bags for 24 hours before rehydration and evaluation (n = 18, in 6 replicates; Fig 1).

### Statistical analysis

Water content data were analyzed by nonlinear regression. Percentage data (except for water contents) were transformed using arcsine transformation before statistical analysis. Digitonin test, trehalose assays for freeze-thaw evaluation and for different trehalose concentrations were analyzed by two-way analysis of variance (ANOVA) followed by Tukey’s multiple test. Data from all the other experiments were assessed by one-way ANOVA, Tukey’s multiple test for mean comparison, and Barlett’s test for homogeneity of the variance. For data that did not pass Barlett’s test, Kruskal-Wallis tests were performed and a two-stage linear step-up procedure of Benjamini, Krieger and Yekutieli method was used to control false discovery rate for post-hoc multiple comparisons. Differences were considered significant at *P* < 0.05 (GraphPad Prism 7.03; GraphPad Software).

## Supporting information

S1 Table The value of fitting parameters in Eq (1) and goodness of fitting (R^2^). (DOCX)

S1 Fig Effect of digitonin treatment and microwave drying on follicular morphology. Two cortical pieces from each replicate were fixed immediately to serve as fresh control. The rest was split into two groups and treated with 0 or 10 μg/ml digitonin for 3 min. Both groups were then exposed to 1.0 M trehalose for 10 min and fixed in Bouin’s solution either before or after 20 min of microwave drying. Follicular morphology was then assessed on tissue sections (n = 35, in 7 replicates). Values are mean ± SD. A significant digitonin treatment-desiccation interaction (P < 0.05) was reported for morphological assessment. # indicates difference (P < 0.05) from all other groups. * represents difference (P < 0.05) between the two treatment groups.
(TIF)

S2 Fig Effect of freeze/thaw on trehalose extraction from ovarian tissue. Four pieces of the digitonin-permeabilized cortical tissues were collected from each ovary (n = 24, in 6 replicates). Two were exposed to trehalose for 10 min and the other two unexposed to serve as controls. Excess trehalose was dabbed off from the tissue with kimwipes. One control and one trehalose-treated pieces were directly immersed in hot water as described in manufacturer’s instruction. The others were snap frozen and then thawed to disrupt cell membrane before trehalose extraction. Trehalose assays were then performed to measure trehalose content in the tissue. Values are mean ± SEM. Values with different letters differ (P < 0.05).
(TIF)

S3 Fig Effect of drying time and storage on follicle survival in the absence of trehalose. Two cortical pieces from each replicate were fixed immediately to serve as fresh control. The other two pieces were stored immediately at 4°C. The rest was exposed to digitonin and TE buffer and microwave dried for 0, 5, 10, or 20 min before 4°C storage. After 1-day storage, tissues were rehydrated and evaluated for follicle survival (n = 36, in 6 replicates). Values are mean ± SD. Values with different letters differ (P < 0.05).
(TIF)
