# Supplementary material for: Influence of microwave-assisted dehydration on morphological integrity and viability of cat ovarian tissues: First steps toward long-term preservation of complex biomaterials at supra-zero temperatures
Source: PLoS One. 2019 Dec 4;14(12):e0225440. doi: 10.1371/journal.pone.0225440 (PMC6892495; doi:10.1371/journal.pone.0225440)
Supplement: S1 Table — (DOCX) [file pone.0225440.s001.docx]

| Trehalose concentration | | 0 M | 0.2 M | 0.5 M | 1.0 M |
| --- | --- | --- | --- | --- | --- |
| Water weight | *W_0_* | 738.9 ± 35.8 | 663.3 ± 29.7 | 418.4 ± 21.2 | 316.5 ± 16.5 |
|  | *W_e_* | 36.5 ± 21.5 | 23.8 ± 15.3 | 29.5 ± 9.8 | 54.4 ± 6.9 |
|  | *k* | 0.1309 ± 0.0118 | 0.1622 ± 0.0197 | 0.2053 ± 0.03 | 0.2973 ± 0.0582 |
|  | *R^2^* | 0.865 | 0.887 | 0.851 | 0.810 |
| Water percentage | *W_0_* | 91.0 ± 3.4 | 81.7 ± 2.1 | 71.0 ± 1.9 | 54.1 ± 2.1 |
|  | *W_e_* | 5.7 ± 2.1 | 3.9 ± 1.0 | 5.7 ± 0.8 | 8.6 ± 0.9 |
|  | *k* | 0.1279 ± 0.0139 | 0.1845 ± 0.0131 | 0.2637 ± 0.7893 | 0.27 ± 0.0366 |
|  | *R^2^* | 0.91 | 0.959 | 0.955 | 0.889 |

**S1 Table.** The value of fitting parameters in Equation (1) and goodness of fitting (R^2^).
