# Supplementary figures and images for: Influence of microwave-assisted dehydration on morphological integrity and viability of cat ovarian tissues: First steps toward long-term preservation of complex biomaterials at supra-zero temperatures
Source: PLoS One. 2019 Dec 4;14(12):e0225440. doi: 10.1371/journal.pone.0225440 (PMC6892495; doi:10.1371/journal.pone.0225440)

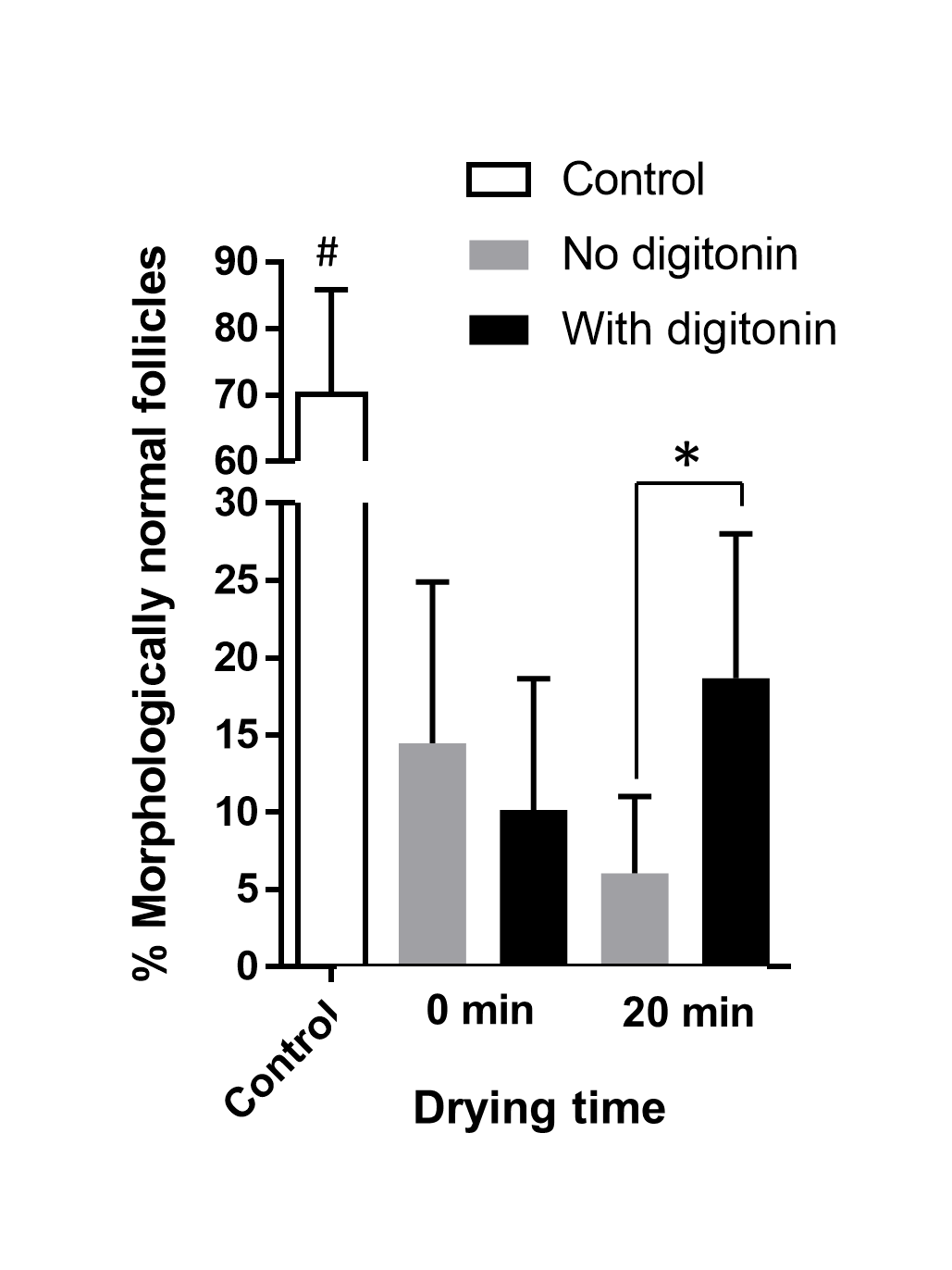

Supplement: S1 Fig — Two cortical pieces from each replicate were fixed immediately to serve as fresh control. The rest was split into two groups and treated with 0 or 10 μg/ml digitonin for 3 min. Both groups were then exposed to 1.0 M trehalose for 10 min and fixed in Bouin’s solution either before or after 20 min of microwave drying. Follicular morphology was then assessed on tissue sections (n = 35, in 7 replicates). Values are mean ± SD. A significant digitonin treatment-desiccation interaction (P < 0.05) was reported for morphological assessment. # indicates difference (P < 0.05) from all other groups. * represents difference (P < 0.05) between the two treatment groups. (TIF) [file pone.0225440.s002.tif]

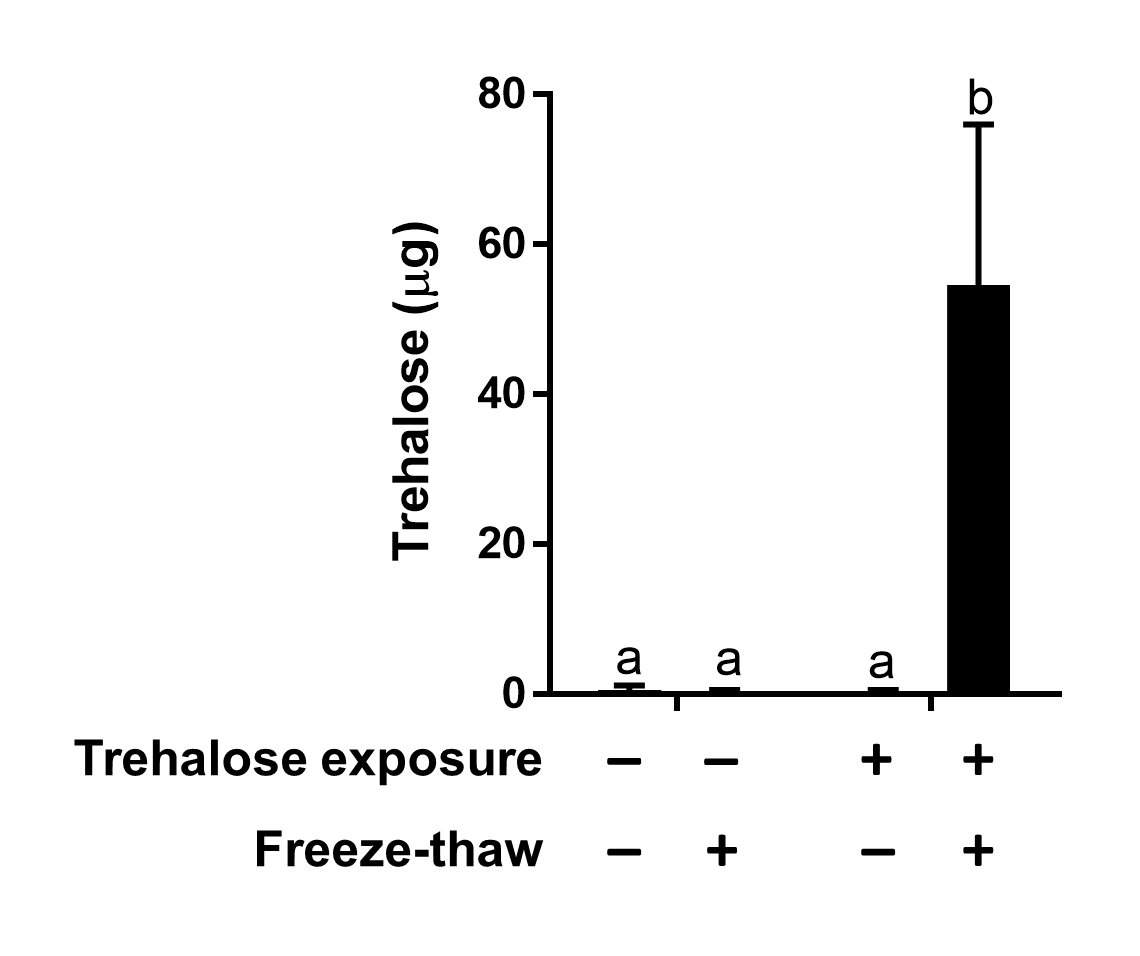

Supplement: S2 Fig — Four pieces of the digitonin-permeabilized cortical tissues were collected from each ovary (n = 24, in 6 replicates). Two were exposed to trehalose for 10 min and the other two unexposed to serve as controls. Excess trehalose was dabbed off from the tissue with kimwipes. One control and one trehalose-treated pieces were directly immersed in hot water as described in manufacturer’s instruction. The others were snap frozen and then thawed to disrupt cell membrane before trehalose extraction. Trehalose assays were then performed to measure trehalose content in the tissue. Values are mean ± SEM. Values with different letters differ (P < 0.05). (TIF) [file pone.0225440.s003.tif]

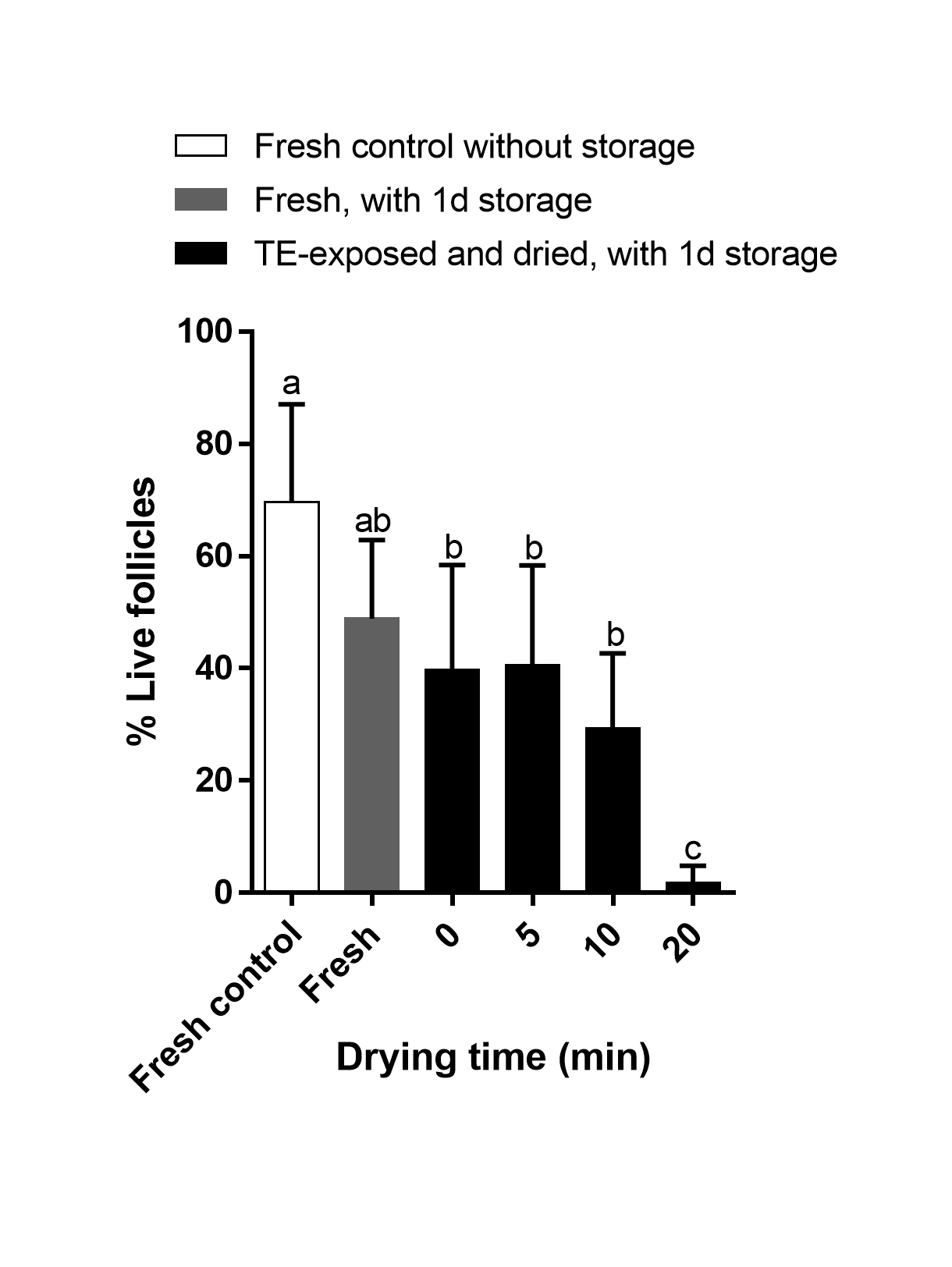

Supplement: S3 Fig — Two cortical pieces from each replicate were fixed immediately to serve as fresh control. The other two pieces were stored immediately at 4°C. The rest was exposed to digitonin and TE buffer and microwave dried for 0, 5, 10, or 20 min before 4°C storage. After 1-day storage, tissues were rehydrated and evaluated for follicle survival (n = 36, in 6 replicates). Values are mean ± SD. Values with different letters differ (P < 0.05). (TIF) [file pone.0225440.s004.tif]
